# Kinematic Analysis Quantifies Gait Abnormalities Associated with Lameness in Broiler Chickens and Identifies Evolutionary Gait Differences

**DOI:** 10.1371/journal.pone.0040800

**Published:** 2012-07-17

**Authors:** Gina Caplen, Becky Hothersall, Joanna C. Murrell, Christine J. Nicol, Avril E. Waterman-Pearson, Claire A. Weeks, G. Robert Colborne

**Affiliations:** 1 School of Veterinary Sciences, University of Bristol, Bristol, United Kingdom; 2 Centre for Comparative and Clinical Anatomy, University of Bristol, Bristol, United Kingdom; University of Hong Kong, China

## Abstract

This is the first time that gait characteristics of broiler (meat) chickens have been compared with their progenitor, jungle fowl, and the first kinematic study to report a link between broiler gait parameters and defined lameness scores. A commercial motion-capturing system recorded three-dimensional temporospatial information during walking. The hypothesis was that the gait characteristics of non-lame broilers (n = 10) would be intermediate to those of lame broilers (n = 12) and jungle fowl (n = 10, tested at two ages: immature and adult). Data analysed using multi-level models, to define an extensive range of baseline gait parameters, revealed inter-group similarities and differences. Natural selection is likely to have made jungle fowl walking gait highly efficient. Modern broiler chickens possess an unbalanced body conformation due to intense genetic selection for additional breast muscle (pectoral hypertrophy) and whole body mass. Together with rapid growth, this promotes compensatory gait adaptations to minimise energy expenditure and triggers high lameness prevalence within commercial flocks; lameness creating further disruption to the gait cycle and being an important welfare issue. Clear differences were observed between the two lines (short stance phase, little double-support, low leg lift, and little back displacement in adult jungle fowl; much double-support, high leg lift, and substantial vertical back movement in sound broilers) presumably related to mass and body conformation. Similarities included stride length and duration. Additional modifications were also identified in lame broilers (short stride length and duration, substantial lateral back movement, reduced velocity) presumably linked to musculo-skeletal abnormalities. Reduced walking velocity suggests an attempt to minimise skeletal stress and/or discomfort, while a shorter stride length and time, together with longer stance and double-support phases, are associated with instability. We envisage a key future role for this highly quantitative methodology in pain assessment (associated with broiler lameness) including experimental examination of therapeutic agent efficacy.

## Introduction

The most widely used methodology for evaluating lameness within commercial broiler flocks is the Bristol six-point ‘gait scoring’ scale developed by Kestin et al [1], whereby trained personnel assign a gait score (GS) between GS0 (normal) and GS5 (cannot walk) based upon a broad range of criteria. The system is well suited for on-farm welfare assessment since it is quick and requires no specialised equipment. However, as a subjective methodology it suffers from drawbacks as a research tool, namely a lack of capacity to discriminate lameness type and an inability to identify the specific gait parameter(s) affected. Kinetic (the measure of forces involved in walking) and kinematic (the study of body motion) methodologies offer quantitative means of gait assessment. Both are regularly used in mammalian veterinary medicine to assess lameness and evaluate surgical and medical treatments [2]. Kinetic data have been collected from poultry using a variety of techniques, with varying measures of success, including the pedobarograph [3], force plate [4], [5], and piezoelectric pressure-sensing mat [6]. Of these studies only one [5] has attempted to link kinetic measures with broiler lameness (as defined by gait score); however, high levels of unsuitable data resulted in many birds having only one or two steps analysed. Constant walking speed is extremely important when using a force-plate due to the restricted size of the data collection surface (compared to a traditional runway); a bird pausing or sitting can discount a ‘run’ of data. The generation of background ‘noise’ can also be problematic, and retrospective data processing is often required to ascertain data suitability. The collection of kinematic data from poultry is less well documented (e.g. [7]–[10]), most studies utilising primitive methodologies (e.g. two-dimensional analysis of videotaped data). We consider kinematic analysis to have real potential for studying poultry gait since the technique benefits from a wide number of possible measures (maximising the likelihood for identifying inter-group differences), allows rapid assessment of data suitability, and facilitates the selection and collection of useful data from lame birds even if they pause or sit halfway through a run. The development of computer assisted three-dimensional kinematic gait analysis now allows comprehensive definition of gait characteristics over a range of speeds to enable accurate comparisons between experimental groups. This study is the first to compare (by any methodology) the gait characteristics of broiler chickens with their ancestral line (red jungle fowl), and the first kinematic study to report a link between broiler gait parameters and defined lameness scores.

Over the last 50 years broiler (meat) chickens have been subjected to intense genetic selection for increased growth rate and body mass; growth rates having risen by over 300% (from 25 to 100 g per day) [11]. In 1950 broilers took 16 weeks to reach marketable weight [12], compared to <40 days in most modern commercial strains. The post-hatch mass of the Red Jungle Fowl (*Gallus gallus*) is approximately 300 g at 35 days [13], compared to 1046 g for a traditional strain of domestic chicken, and 1800 g for Ross broilers [12]. Selection has specifically targeted the breast muscle, producing a condition termed pectoral hypertrophy. Approximately 18% of Ross body mass is breast muscle, compared to 9% in both jungle fowl and a traditional strain [12], [13]. In addition, a greater thigh muscle and leg bone mass, and relatively short legs [14], distinguishes modern broiler strains from the ancestral line.

The walking gaits of terrestrial bipeds (e.g. birds and humans) have naturally evolved energy efficiency. At constant walking speed the bipedal body behaves as an inverted pendulum. The centre of mass (CoM) rises over each supporting (stance-phase) leg in turn, decelerating and accelerating with each step, alternating transfer between potential and kinetic energy. The biomechanics of bipedal gait, including efficiencies and energetic fluctuation, are described elsewhere [15], [16]. Although no-one had previously compared the gait characteristics of broilers with the ancestral subspecies we hypothesised that the gait characteristics of the jungle fowl, the progenitor to all modern breeds, would be ‘optimal’ (i.e. symmetrical, low amplitude, vertical and lateral displacement of the CoM) in relation to domestic strains.

Intensive production is associated with a high prevalence of lameness; almost 30% of broilers assessed in a recent UK survey had substantial gait abnormalities [11]. Lameness indicates an interruption of the ‘normal’ gait cycle and, thereby, increases energy expenditure [17], [18]. Lameness can take many forms; it can be of infectious or non-infectious (e.g. developmental or degenerative) origin, and can involve tendons, joints, ligaments, and bones [19], [20]. Strong correlations between body mass, growth rate, and lameness have been documented [21], [22], although there is little evidence to link the severity of lameness with pathology [5], [23]–[25]. Since gait patterns appear to be linked to species morphology [26] differences in gait between modern broilers and their ancestral line will likely be due to a combination of greater body mass, abnormal morphology, and often (but not necessarily) pathology.

The avian CoM is normally located well forward of the hip due to the horizontal orientation of the vertebral column [27]; however, pectoral hypertrophy causes physical unbalance by displacing the CoM cranially [26]. Increasing body mass places greater demands on the immature broiler skeleton, while the change in body shape (displaced CoM, reduced leg length etc) requires the leg muscles to generate greater biomechanical forces (and stress) during locomotion [27], [28]. In response to an injury, heavy load, or unbalanced posture, an animal will perform compensatory gait adaptations to minimise the additional energy expenditure required for movement [28]. Consequently, the gait patterns of heavy meat-type poultry differ from the lighter egg-type strains (e.g. [9]), with certain modifications such as wide stance, and hip and foot rotation, considered likely to lead to further progressive problems [29].

The purpose of this study was to utilise a commercially available system to record temporo-spatial gait data from a modern broiler strain and jungle fowl (the ancestral line) while walking at an unrestrained speed. The use of multi-level modelling allowed comparisons to be made between the two sub-species at analogous walking velocities, and the additional effect of moderate lameness (GS3) on broiler gait to be investigated. Data were collected from the same group of jungle fowl at two ages (immature and adult) to allow comparison with the broilers at both a comparable age and body size. The hypothesis tested whether the gait patterns recorded for non-lame (GS0) broilers were intermediate to those recorded for lame broilers and the jungle fowl, initial differences in broiler gait being influenced by growth rate and body conformation, and additional modifications linked to leg weakness.

## Materials and Methods

### Statement of Ethical Approval

This project was carried out following ethical approval by the University of Bristol (Home Office Licence PPL30/2865).

### Animals and Husbandry

Groups of 32-day old, mixed sex, standard-reared broiler chickens were selected from a commercial flock four days prior to data collection. These groups consisted of either mildly lame (GS2) or non-lame (GS0) birds. To standardise the GS2 group as much as possible birds without any obvious unilateral gait impairment (limp), or pronounced skeletal deformities such as valgus/varus, were selected. Following transport to the research facility birds were housed in groups of twelve within pens (3×1.5 m) on wood shavings, within an ambient temperature of 20°C, and a 16L: 8D light-cycle. They had access to water and feed *ad-libitum* (with the exception of two 2 h periods of food withdrawal on the day of testing). Diet (commercial grower pellets) was obtained from the same farm as the birds. Following a day of rest, all birds were weighed, examined, and gait scored daily to monitor their welfare. Any bird that demonstrated signs of consistent visible distress (e.g. abnormal vocalisation), illness, or reached GS4 (‘a severe gait defect, only walking, with difficulty, when driven’, [1]) was removed from the study.

Captive-bred jungle fowl were obtained at age 4 weeks and housed in groups of seven (male) or six (female) in 3×3 m pens under conditions similar to the broilers. Diet comprised layer’s mash supplemented with fresh fruit and vegetables.

### Visual Assessment of Walking Ability

The gait score of each bird was agreed by two experienced assessors using the Bristol system (see [1]). The initial selection criterion of GS2 was ‘an identifiable gait defect that had little impact upon mobility, typically taking the form of a straight-legged kicking gait’. Moderately lame (GS3) birds had ‘obvious gait defects that affected their ability to move’. Since the jungle fowl (JF) formed the control non-lame group they were only gait-scored once at the beginning of the trial and once at the end to ensure that they had maintained leg health (GS0).

### Kinematic Data Collection

Birds were tested in separate batches according to group. Lame birds that reached GS3 were tested at age 39 days (n = 12), the non-lame GS0 birds (same strain) were tested at age 40 days (n = 10), while the jungle fowl (n = 10) were tested at 8 weeks old (JF_1_, immature) and again when 12 months old (JF_2_, adult).

#### Training

**Figure 1:**
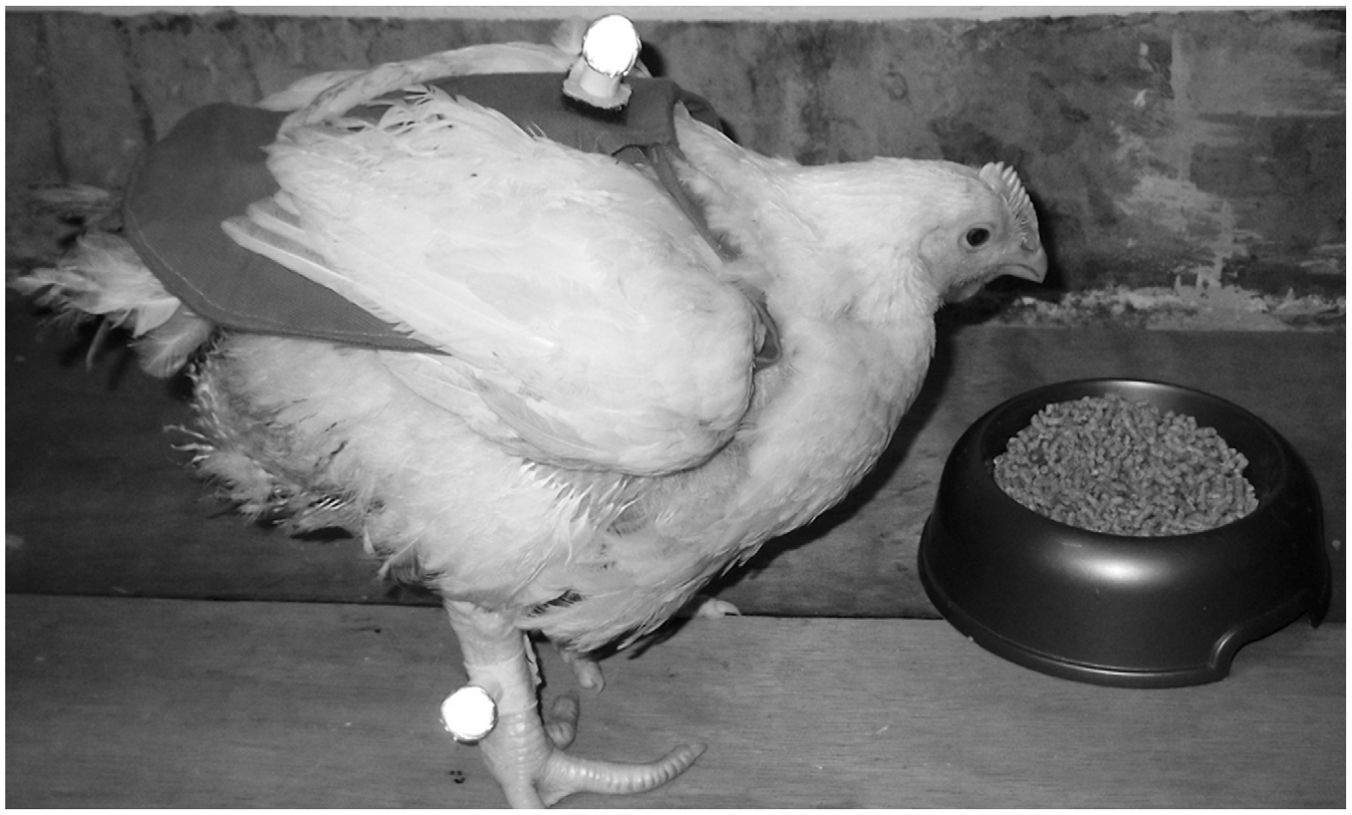
Position of reflective markers for kinematic data collection from a broiler chicken.

For two days prior to testing all potential test birds were acclimatised to wearing a poultry saddle (that did not interfere with wing mobility) and Lycra leg-bands (*Figure 1*). Poultry saddles are commercially available and prevent skin damage to female backs (by the cockerel) during mating. On the day prior to testing, birds that tolerated wearing the saddle were trained within the test environment to walk towards a bowl containing a feed reward (mealworms and broiler pellets) and a social cue. The addition of a conspecific social cue was required as individuals varied in food motivation; this took the form of two pen-mates positioned at the end of the runway behind a wide-gauge plastic net partition. Only birds that consistently walked towards the cues during the training sessions were used for data collection.

#### Testing

Each group of birds was tested on a separate day. Two hours prior to testing all food was withdrawn from the home pens. The final group of test broilers was determined by gait scoring, and these and the jungle fowl were fitted with poultry saddles and leg bands within their home pen. Spherical retro-reflective markers and an infra-red four-camera Qualisys ProReflex® motion capturing system (Qualisys AB, Gothenburg, Sweden), were used to collect three dimensional (3D) kinematic data using Qualisys Track Manager (QTM) software (Qualisys AB). Two central cameras were positioned facing the runway at a height of approximately 1.0 m, and two further cameras were positioned either side of these at a height of approximately 0.4 m. Three markers were attached to each bird immediately prior to filming: one (15 mm diameter) was attached to the mid-line of the saddle at the base of the neck, and a further two (10 mm diameter) were attached to the posterior aspect of the metatarsal bone, immediately above each foot, using Lycra bands. Each marker was stabilised against wobble with the addition of a small plastic ring at the base (*Figure 1*). The saddles (and back markers) worn by the different groups differed in size/weight as follows: 8 g (JF_1_), 14 g (JF_2_), and 28 g (GS0, GS3). This was equivalent to 1.8% (JF_1_), 1.3% (JF_2_), 1.5% (GS0), and 1.1% (GS3) of their mean total body mass. All groups wore the same leg markers, weighing 3 g each. This was equivalent to an additional 1.4% (JF_1_), 0.5% (JF_2_), 0.3% (GS0), and 0.2% (GS3) of their mean total body mass.

**Figure 2:**
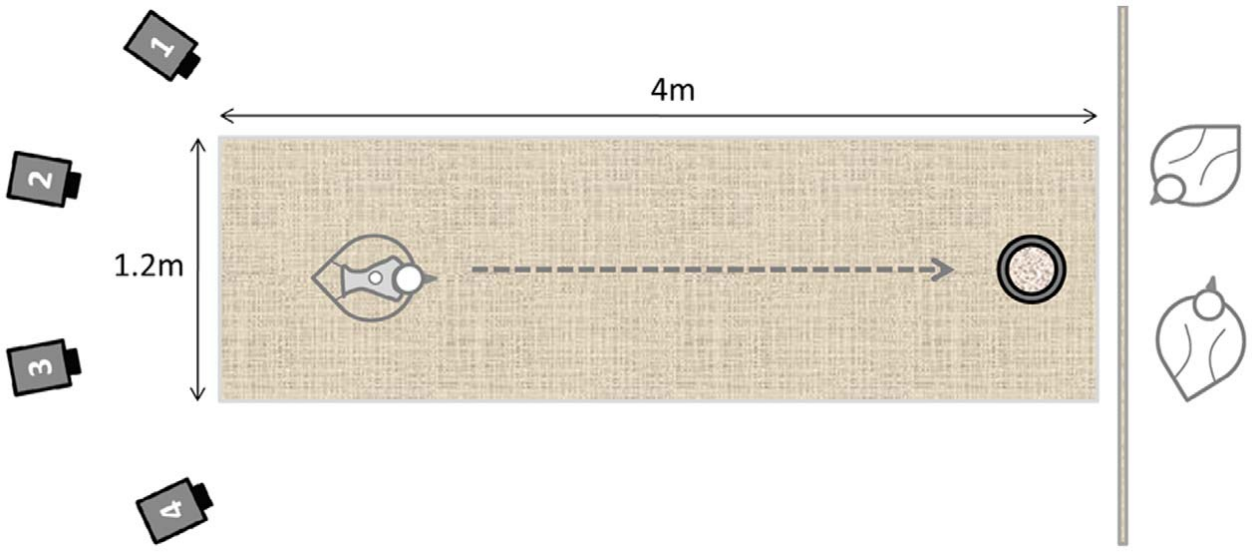
Plan view of the runway set-up to capture kinematic gait data from test birds. Four Qualisys ProReflex® cameras were aimed down the runway at the test bird (wearing reflective markers) located within the calibrated space. The test bird is located in the start position, facing a food reward and social cue (provided by two pen mates partitioned behind a net screen).

The birds walked along a 3 m runway, contained within a calibrated space of 0.8×1.2×4 m (h×w×l), towards the food reward and social cue as in training (*Figure 2*). Once a bird began to walk the system was triggered manually and the cameras simultaneously filmed for 15 s at 120 Hz, tracking the markers in 3D space. The aim was to collect six successful runs from each bird, (a ‘run’ being a single continuous passage along at least half of the runway at a near-constant speed), but this depended upon the motivation of each particular bird to walk; some birds facilitated the collection of more data, others less. Following data collection the saddle and leg markers were removed and the bird was returned to its home pen.

### Data Processing

**Figure 3:**
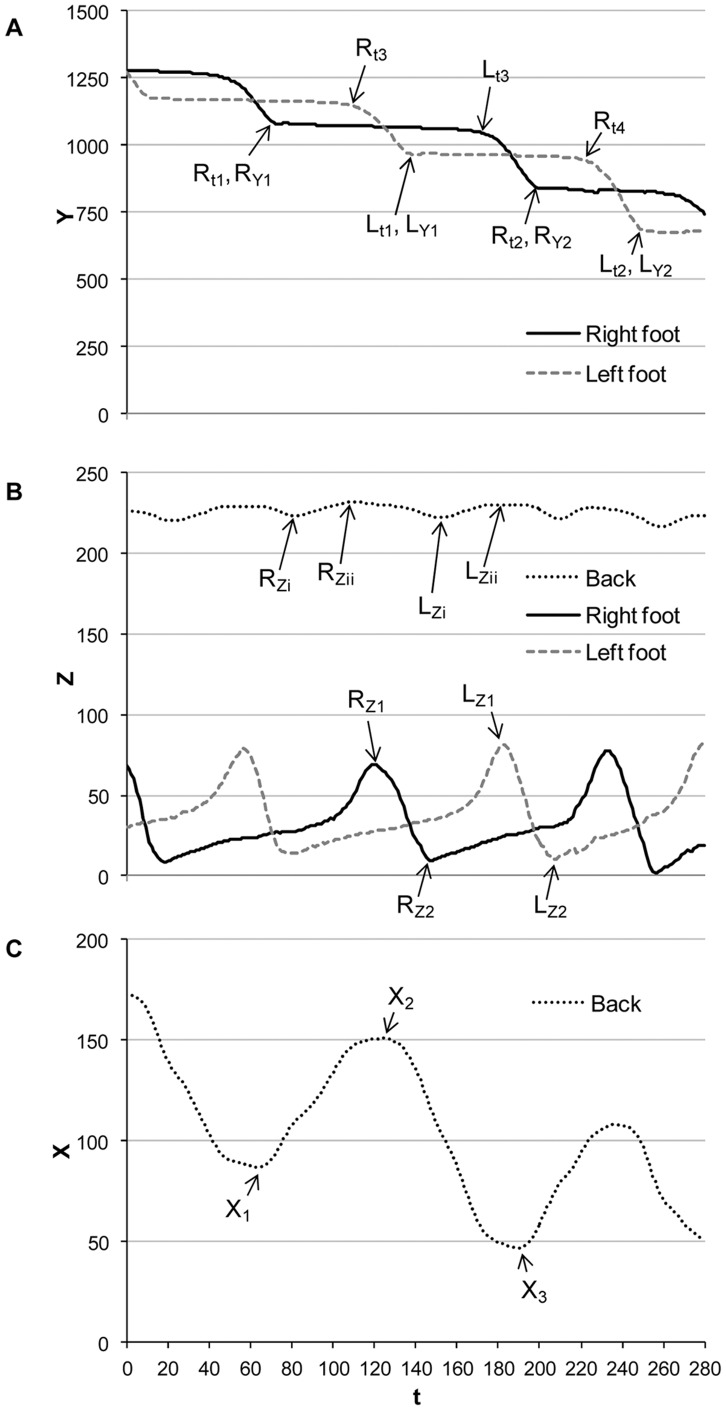
Example of a typical segment of kinematic data illustrating the spatial and temporal progression of the reflective markers from a single broiler as it moved along the runway. (a) Alternating right (R) and left (L) steps with (flat) stance and swing phases evident. The y co-ordinate (y-axis) represents the craniocaudal location of the leg marker spatially (‘0′ being the mid-point of the runway), while the x-axis represents time (t); (b) vertical back (VB) and vertical leg (VL) displacement; (c) lateral back displacement (LB) viewed from above. The letters with subscripts (R,L and X) denote examples of reference points that are used in calculating the various kinematic variables (as defined in *Table 1*).

The QTM software assigned numerical values to the x (transverse), y (craniocaudal), and z (vertical) co-ordinates for each marker in 3D space. Quantitative analysis of this raw data set was performed in Excel. *Figure 3* illustrates an example segment of kinematic data illustrating the spatial and temporal progression of the reflective markers from a single bird as it moved along the runway. From these plots a set of kinematic parameters relating to basic gait characteristics were calculated for each suitable stride. Gait parameters included: stride duration (SD), stride length (SL), percentage stance (ST), double-leg support (DS), vertical leg displacement (VL), lateral back displacement (LB), vertical back displacement (VB), and velocity (VEL) (*Table 1*). The gait parameters SL, VL, LB, VB and VEL were normalised for hip height, since it was not possible to balance the groups for this measure. This ensured that the comparison between shorter and longer legged individuals would be valid. Relative values of SL, VL, LB, and VB were calculated by dividing the absolute value by hip height (mm) at the middle of the stance phase. Relative VEL was calculated by dividing ‘relative SL’ by SD.

**Table 1:** Calculations for kinematic variables (gait parameters) using the example reference points detailed in *Figure 3*.

Gait Parameter (unit)	Description	Right leg (R)	Left leg (L)
SD (s)	Time taken to complete one stance (groundcontact), and one swing (aerial), phase	SD_R_ = (R_t2_–R_t1_)/120	SD_L_ = (L_t2_–L_t1_)/120
SL (mm)	Distance moved during the stance andswing phase of a single leg	SL_R_ = R_Y2_–R_Y1_	SL_L_ = L_Y2_–L_Y1_
ST (%)	Percentage of the stride duration when afoot is in contact with the ground	ST_R_ = (R_t3_–R_t1_)/SD_R_	ST_L_ = (L_t3_–L_t1_)/SD_L_
DS (%)	Percentage duration of each stride whenboth legs are weight-bearing	DS_R_ = [(R_t3_–L_t1_)/(R_t2_–R_t1_)]*100	DS_L_ = [(L_t3_–R_t4_)/(L_t2_–L_t1_)]*100
VL (mm)	Maximum height leg liftedduring a stride	VL_R_ = R_Z1_–R_Z2_	VL_L_ = L_Z1_–L_Z2_
LB (mm)	Maximum lateral (side-to-side) backmovement recorded during a stride	LB_R_ = X_2_–X_1_	LB_L_ = X_2_–X_3_
VB (mm)	Maximum height back moved in a verticaldirection during a stride	VB_R_ = R_Zii_–R_Zi_	VB_L_ = L_Zii_–L_Zi_
VEL (mm/s)	Speed in a given direction	VEL_R_ = SL_R_/SD_R_	VEL_L_ = SL_L_/SD_L_

SD = stride duration, SL = stride length, ST = stance, DS = double-leg support, VL = vertical leg displacement, LB = lateral back displacement, VB = vertical back displacement, VEL = velocity.

Measurements for each gait parameter were collected in paired over-lapping strides (one right and one left) and then averaged to produce mean values. Strides were deemed ‘suitable’ for analysis only if they had data available for all three markers in each dimension. Strides were only analysed from the middle section of any run; those from the very beginning (acceleration) and those from the end (deceleration) were excluded. Due to variability in bird performance the repeated-measures data set generated was unbalanced. Group mean ± S.D. values were calculated using individual mean values for all parameters and the coefficient of variation (%CV) for each calculated as an estimate of inter-individual variation [%CV = (S.D./mean)*100].

### Statistics

Multilevel modelling software MLwiN v2.22 was used to create random-intercept nested models reflecting the hierarchical structure of the raw data set, whereby the gait parameter of interest was selected as the response variable, the nested hierarchy comprised three levels (stride, run, and bird ID), and ‘group’ (GS0, GS3, JF_1_, JF_2_) was the primary predictor. For those gait parameters where VEL was included as an additional predictor (linear, 2^nd^, or 3^rd^ degree polynomial) a first-order interaction between ‘group’ and VEL was also added to the model. Prior to analysis standardised residuals were calculated and plotted against normal scores for all models and any obvious outliers were selected and omitted from the analysis. To detect whether a predictor was having a significant effect within a model the relevant coefficient (Coeff) and standard error of coefficient (SE_coeff_) values were used to calculate respective p-values.

## Results

### Physical Differences

There were significant differences in body mass between the groups. Immature jungle fowl (JF_1_) were significantly lighter than adult jungle fowl (JF_2_) (JF_1_, mean ± SD: 0.44±0.06 kg, n = 10, JF_2_, 1.08±0.23 kg, n = 10, paired-samples T-test: t_1,9_ = 7.34, p<0.001). Tukey post-hoc comparisons revealed that the JF_1_ were also significantly lighter than the non-lame (GS0∶1.84±0.13 kg, n = 10), and lame (GS3∶2.49±0.17 kg, n = 12) broilers (one-way ANOVA: *F*_(2,29)_ = 668.97, *p*<0.001). The JF_2_ were also significantly lighter than either broiler group and the GS0 were significantly lighter than GS3 (one-way ANOVA: *F*_(2,29)_ = 165.13, *p*<0.001). When body mass was included within the models as an additional predictor (covariate) it had no significant effect on any gait parameter, presumably as the marked variation between ‘group’ had already been accounted for.

Differences in hip height were also apparent between the groups. The JF_1_ had significantly shorter hip height than the JF_2_ (JF_1_, 175.20±8.27 mm, JF_2_, 227.37±22.57 mm, paired-samples T-test: t_1,9_ = 5.50, p<0.001). Tukey post-hoc comparisons revealed that the JF_1_ also had significantly shorter hip height than the GS0 (225.19±13.96 mm) and the GS3 (232.89±14.40 mm) (one-way ANOVA: *F*_(2,29)_ = 62.69, *p*<0.001). JF_2_, GS0, and GS3 had comparable hip height (one-way ANOVA: *F*_(2,29)_ = 0.591, *p* = 0.560).

### Gait Parameters

#### Inter-individual variation

Untransformed mean group values (± S.D.) and %CV for each gait parameter are provided within *Table 2*. JF_2_ had the least variation of the four groups for all gait parameters except DS, for which it had the most. Of the four groups JF_1_ had the greatest variation in LB and VEL, and the least variation in DS. GS0 had the most variation in SD, SL, ST, and VL, while GS3 had the most variation in VB. For all gait parameters except DS, VB, and VEL, lame broilers demonstrated more inter-individual variation than the non-lame broilers.

**Table 2:** Group mean, S.D., and coefficient of variation (%CV) values for a series of gait parameters (calculated using individual mean values): jungle fowl (immature, JF_1_; adult, JF_2_, n = 10), non-lame broilers (GS0, n = 10) and lame-broilers (GS3, n = 12).

Gait parameter	JF_1_		JF_2_		GS0		GS3	
	Mean (± SD)	%CV	Mean (± SD)	%CV	Mean (± SD)	%CV	Mean (± SD)	%CV
SD (s)	0.58	19.5	0.77	15.9	0.63	22.9	0.60	22.2
	(±0.11)		(±0.12)		(±0.14)		(±0.13)	
SL (mm)	385.25	13.3	401.19	7.1	513.96	20.7	373.46	17.1
	(±51.14)		(±28.31)		(±106.60)		(±63.74)	
ST (%)	53.54	8.8	56.35	4.0	52.46	13.5	58.93	8.3
	(±4.72)		(±2.27)		(±7.06)		(±4.91)	
DS (%)	14.56	15.9	13.37	35.1	21.29	27.0	18.72	34.7
	(±2.32)		(±4.70)		(±5.75)		(±6.49)	
VL (mm)	64.25	10.0	57.63	8.0	99.41	10.3	89.00	9.5
	(±6.44)		(±4.59)		(±10.19)		(±8.44)	
LB (mm)	25.97	27.4	26.70	23.3	71.07	24.2	82.93	23.7
	(±7.11)		(±6.23)		(±17.19)		(±19.62)	
VL (mm)	11.31	45.3	6.41	21.9	24.75	22.4	20.29	47.6
	(±5.13)		(±1.41)		(±5.54)		(±9.66)	
VEL (mm/s)	777.48	37.8	562.76	19.9	1006.48	35.5	709.99	36.5
	(±294.17)		(±111.96)		(±357.38)		(±259.38)	

SD = stride duration, SL = stride length, ST = stance, DS = double-leg support, VL = vertical leg displacement, LB = lateral back displacement, VB = vertical back displacement, VEL = velocity.

#### Stride Duration (SD)

**Figure 4:**
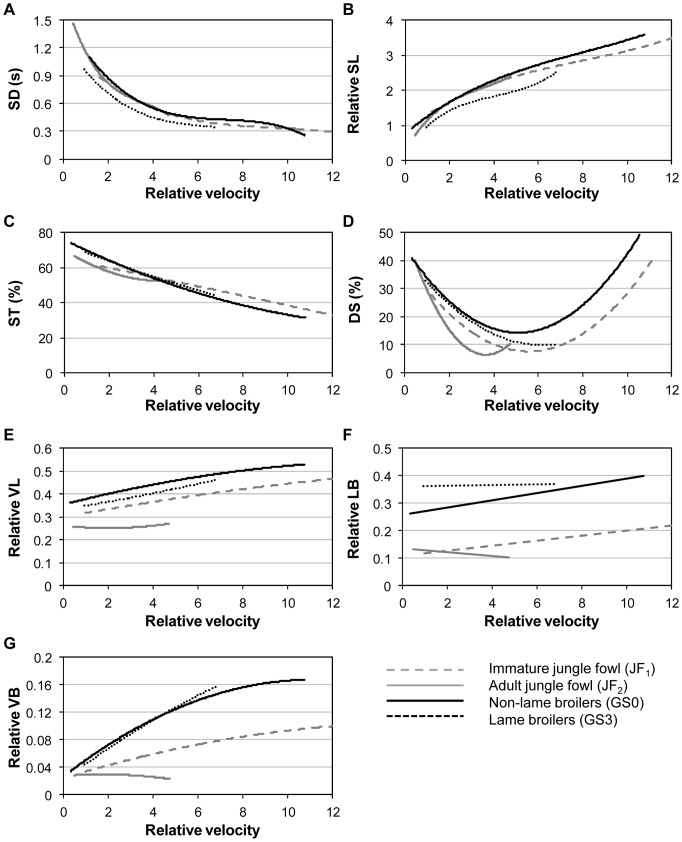
Differences in gait parameter between four avian groups. (a) stride duration, SD, (b) relative stride length, SL, (c) percentage stance, ST, (d) double-leg support, DS, (e) relative vertical leg displacement, VL, (f) relative lateral back displacement, LB, (g) relative vertical back displacement, VB.

SD decreased with increasing relative velocity in all groups (*Figure 4a*). The rate of decline was greater at slower speeds, dropping to a plateau at higher speeds. The jungle fowl (both ages) took strides of comparable duration to the GS0 broilers, while the GS3 broilers took significantly faster strides (*Table 3*).

**Table 3:** Differences in gait parameter (Gait) between jungle fowl, (juvenile, ‘JF_1_’, and mature, ‘JF_2_’, n = 10), non-lame broilers, ‘GS0’ (n = 10), and lame broilers, ‘GS3’ (n = 12).

Gait	n	VEL_poly_	Group	JF_2_		GS0		GS3	
				Coeff (SE_coeff_)	P	Coeff (SE_coeff_)	P	Coeff (SE_coeff_)	P
SD	610	3	JF_1_	0.011 (0.021)	ns	0.000 (0.020)	ns	−0.109 (0.020)	*0.000*
			JF_2_	-	-	−0.011 (0.022)	ns	−0.120 (0.022)	*0.000*
			GS0	-	-	-	-	−0.109 (0.020)	*0.000*
SL^a^	615	3	JF_1_	−0.025 (0.068)	ns	0.060 (0.064)	ns	−0.414 (0.064)	*0.000*
			JF_2_	-	*-*	0.085 (0.069)	ns	−0.329 (0.068)	*0.000*
			GS0	-	-	-	-	−0.414 (0.064)	*0.000*
ST	604	2	JF_1_	−2.630 (1.238)	*0.033*	0.122 (1.127)	ns	0.832 (1.178)	ns
			JF_2_	-	-	2.752 (1.262)	*0.029*	3.462 (1.284)	*0.007*
			GS0	-	-	-	-	0.710 (1.178)	ns
DS	612	2	JF_1_	−5.041 (2.099)	*0.016*	5.297 (1.621)	*0.001*	3.726 (1.943)	0.054
			JF_2_	-	-	10.338 (2.186)	*0.000*	8.768 (2.435)	*0.000*
			GS0	-	-	-	-	−1.571 (2.037)	ns
VL^a^	613	2	JF_1_	−0.102 (0.016)	*0.000*	0.074 (0.016)	*0.000*	0.036 (0.015)	*0.016*
			JF_2_	-	-	0.176 (0.016)	*0.000*	0.138 (0.016)	*0.000*
			GS0	-	-	-	-	−0.038 (0.015)	*0.011*
LB^a^	614	1	JF_1_	−0.033 (0.028)	ns	0.165 (0.026)	*0.000*	0.224 (0.026)	*0.000*
			JF_2_	-	-	0.197 (0.028)	*0.000*	0.257 (0.027)	*0.000*
			GS0	-	-	-	-	0.060 (0.026)	*0.021*
VB^a^	611	2	JF_1_	−0.030 (0.007)	*0.000*	0.047 (0.006)	*0.000*	0.045 (0.006)	*0.000*
			JF_2_	-	-	0.077 (0.007)	*0.000*	0.075 (0.008)	*0.000*
			GS0	-	-	-	-	−0.002 (0.006)	ns
VEL^a^	614	-	JF_1_	−1.956 (0.618)	*0.001*	0.415 (0.609)	ns	−1.222 (0.386)	*0.042*
			JF_2_	-	-	2.371 (0.620)	*0.027*	0.734 (0.614)	ns
			GS0	-	-	-	-	−1.636 (0.605)	*0.007*

SD = stride duration, SL = stride length, ST = stance, DS = double-leg support, VL = vertical leg displacement, LB = lateral back displacement, VB = vertical back displacement, VEL = velocity. VEL_poly_ = the order of polynomial degree attributed to relative velocity within the model. The coefficient (Coeff) gives the amount of change in measure (gait parameter) for a unit change in each variable (Group). A positive coefficient estimate indicates that an increase in the value of a variable is associated with an increase in the respective measure and a negative coefficient estimate indicates a decrease.

a Normalised relative to hip height.

#### Stride Length (SL)

Relative SL increased with relative velocity in all groups (*Figure 4b*). Relative SL values for the GS0 broilers and both jungle fowl groups were comparable, and all were significantly greater than the GS3 broilers (*Table 3*).

#### Stance (ST)

ST decreased with relative velocity in all groups (*Figure 4c*), although at a slower rate in the jungle fowl than the broilers. The GS3, GS0, and JF_1_ demonstrated comparable ST. All three spent a significantly greater proportion of each stride in stance than the JF_2_ (*Table 3*), at least until a relative velocity of approximately 4, when the JF_2_ values for ST appeared to plateau.

#### Double-Leg Support (DS)

DS decreased initially with increasing velocity at lower walking speeds (i.e. became a lesser component of the stride). However, at relative velocities above 4 (JF_2_), or 6 (JF_1_, GS0, GS3), DS began to rise again with additional increases in relative velocity (*Figure 4d*), suggesting that DS had reached a minimum value and any further reductions in SD (to further increase walking speed) increased the proportion of the stride spent in DS. Statistically GS0 and GS3 were comparable, GS0 had significantly higher DS than either of the jungle fowl groups, and JF_2_ significantly had the lowest DS (*Table 3*).

#### Vertical Leg Displacement (VL)

Relative VL underwent a pronounced increase with increasing relative velocity in all groups except JF_2_ (*Figure 4e*). The GS0 broilers had the greatest relative VL displacement, significantly more than any other group, while JF_2_ had the least (*Table 3*). In addition, the GS3 broilers lifted their feet significantly higher than the JF_1_.

#### Lateral Back Displacement (LB)

Relative LB increased with increasing relative velocity (*Figure 4f*). The GS3 broilers demonstrated significantly greater LB movement than any other group (*Table 3*). The jungle fowl had significantly lower values than either broiler group; both age groups were comparable.

#### Vertical Back Displacement (VB)

Relative VB increased with velocity in all groups apart from the JF_2_, in which it decreased slightly (*Figure 4g*). The GS0 and GS3 broiler groups were comparable, displaying significantly more relative VB than either jungle fowl group (*Table 3*). The JF_2_ exhibited significantly the least vertical back movement.

#### Velocity (VEL)

The GS0 broilers walked with significantly faster relative velocity than any other group, except JF_1_. The GS3 broilers were significantly slower than JF_1_, but were comparable to JF_2_ (*Table 3*). Values of actual velocity for the four groups, calculated by averaging mean values for each individual (± S.D.), were: GS0, 1.01 (±0.38) m s^−1^, JF_1_, 0.77 (±0.29) m s^−1^, GS3, 0.71 (±0.26) m s^−1^, JF_2_, 0.56 (±0.11) m s^−1^.

### Summary

A summary of the main gait characteristics, ranked, for the four groups is provided in *Table 4*. In brief, when corrected for hip height, the immature jungle fowl (JF_1_) walked quickly, took long-length long-duration strides, with a long stance phase and a medium proportion of double-leg support. The JF_1_ did not lift their feet far off the ground with each stride, and demonstrated little back movement (in both lateral and vertical directions). The adult jungle fowl (JF_2_) walked more slowly, but also took long strides of long duration. They utilised a short stance and double-leg support phase, demonstrated moderate vertical and little lateral back movement, and lifted their legs the least. Non-lame (GS0) broilers walked quickly, taking long-length long-duration strides. They utilised a long stance phase, with high double-leg support. They lifted their feet proportionately the highest distance from the ground with each stride and exhibited much vertical and moderate lateral back movement. The lame broilers (GS3) walked slowly, took short quick strides, and utilised a long stance phase with high double-leg support. They lifted their legs moderately high and demonstrated high levels of back movement in both planes.

**Table 4:** Summary of gait characteristics and ranking^a^ (according to significance testing, *Table 3*, and data models, *Figure 4*) for four avian groups: immature jungle fowl (JF_1_), adult jungle fowl (JF_2_), non-lame broilers (GS0), and lame broilers (GS3).

Gait Parameter	JF_1_	JF_2_	GS0	GS3
Stride duration (SD)	Slow (1)	Slow (1)	Slow (1)	Fast (2)
Stride length (SL)^b^	Long (1)	Long (1)	Long (1)	Short (2)
Stance (ST)	High (1)	Low (2)	High (1)	High (1)
Double-leg support (DS)	Med (2)	Low (3)	High (1)	High (1)
Vertical leg displacement (VL)^b^	Med-low (3)	Low (4)	High (1)	Med-high (2)
Lateral back displacement (LB)^b^	Little (3)	Little (3)	Med (2)	Much (1)
Vertical back displacement (VB)^b^	Med (2)	Little (3)	Much (1)	Much (1)
Velocity (VEL)^b^	Fast (1)	Slow (2)	Fast (1)	Slow (2)

a Ranking has been assigned using consecutive numbers whereby (1) is the greatest measure and, in cases where all four groups are significantly different, (4) the least. Groups with similar gait parameters have been assigned the same rank.

b Normalised relative to hip height.

## Discussion

### Velocity, Stride Length and Stride Duration

The observation that the non-lame (GS0) broilers walked significantly faster than the lame (GS3) birds was expected, having previously been reported [6], [30]. Reduced speed is hypothesised as being a means of reducing pain and/or decreasing peak vertical force, and thus stress, on the musculoskeletal system [4]. Our overall mean baseline velocity of 0.71 (±0.26) m s^−1^ (GS3, n = 12), was faster than a range of 0.1–0.55 m s^−1^ observed in a previous broiler study [28]. Since the gait score of the birds used in that study was not assessed we are unable to determine whether walking ability was responsible. We hypothesise that the velocity data recorded in the current study is likely to be strongly correlated with appetite; the high speeds attained by both broiler groups were likely to be an effect of the feed withdrawal regime we utilised to encourage birds to approach the food (as determined necessary during pilot observations). Genetic selection for improved feed conversion has lead to increased appetite in broilers [31] which could account for both broiler groups moving at significantly faster speeds than the adult jungle fowl. Jungle fowl also walked faster when immature than when adult, possibly due to higher metabolic requirements when young.

Increased velocity is attained by increasing stride frequency (i.e. the reciprocal of stride duration) and/or the distance travelled with each stride (stride length). Our models demonstrate that the jungle fowl utilised the same tactic for increasing their speed at both ages (i.e. they took strides of comparably long-length and long-duration) (*Figure 4a, b*). In contrast the lame broilers took relatively short strides, and therefore had to reduce their stride duration (increase stride frequency) to accelerate. Decreasing stride duration and length reduces the time that any one leg is in swing, and it is likely that our lame broilers were utilising this mode of walking to increase their stability, especially as they had a greater group mean body mass than the non-lame birds. The enhanced pectoral muscle mass of our heavy lame broilers will have displaced their centre of mass (CoM) cranially, and this would require their feet to be positioned further forward under the body for support. The opposite foot would thus need to be quickly replaced following each step to re-establish and maintain balance [*14*]. Turkeys display shorter stride lengths when lame than when sound [32] and experimentally-induced lameness has been linked to reduced stride length in broilers [33]. Evidence for the short stride characteristic having a substantial morphological component includes the observation that ad-libitum fed intensively-selected broilers took shorter step lengths than either intensively-selected broilers maintained on a restricted diet or an out-bred strain [28]. The expectation that non-lame broilers would take shorter strides than the jungle fowl was, however, not met; our non-lame broiler group took relative stride lengths that were comparable to the jungle fowl at either age. This was especially surprising since the non-lame broilers were significantly heavier than the adult jungle fowl. Although there were no obvious visual signs of chronic inflammation within the leg joints of any lame broiler, an unidentified pathological, and perhaps painful, component might be responsible for producing these altered gait characteristics in addition to their large body mass (the lame broilers also being significantly heavier than the non-lame broilers).

### Stance and Double-leg Support

Stance duration was speed-dependant, decreasing with increasing velocity (the broilers exhibiting a steeper rate of decrease than the jungle fowl). Statistically, as expected, the adult jungle fowl demonstrated the shortest stance phase; however, the lack of any difference between the three other groups was unexpected since lameness is known to affect the stance phase [34]. Extrapolating data collected by Reiter & Bessei [9], from lame and non-lame broilers walking on a treadmill at 0.17 m s^−1^, values of 75% and 42% stance can be calculated respectively, a substantial disparity not observed within our own study.

A modern broiler strain has previously been observed to spend approximately 35% of the cycle in double-leg support [28], a value similarly applicable to all four of our groups at very low walking speeds. Our observation that the broilers had significantly higher double-leg support than the jungle fowl suggests that this modification is a means to maintain balance; broilers having also been shown to demonstrate longer double-leg support phases than layers [9]. Since the period of greatest stress and instability during the bipedal gait cycle occurs during single-limb support it would be beneficial for the broilers to distribute their body mass between the two legs via increased double-leg support times [35]. Less stable humans (e.g. children and the elderly), use shorter swing phases and/or longer stance and double-leg support periods [28]. An increase in stability with age may explain why the immature jungle fowl demonstrated greater double-leg support than the adults. An anticipated increase in double-leg support in the lame broilers (above that seen in non-lame broilers) was not observed.

### Leg and Back Displacement

In broilers the higher leg lift apparent for the non-lame birds appears to be directly linked to stride length since a positive linear relationship (R^2^ = 0.66%) existed between vertical leg displacement and stride length within our data set. The non-lame broilers took longer strides than the lame birds and additional lift would facilitate advanced placement of the foot. Reiter & Bessei [9] observed that broilers with obvious skeletal abnormalities walked with greater and more variable vertical leg lift (6.5±2.0 cm) than sound birds (5.8±0.4 cm) at speeds of 0.17 m s^−1^. However, this is likely to be a direct result of bone deformation, which we avoided when selecting our test group. We hypothesise that the jungle fowl lifted their legs less than the broilers due to greater joint flexibility. Laying hens place their legs directly under the transverse position of the CoM, which enables their body to move in a straight line with little lateral displacement [9], and the jungle fowl employed a similar mode of walking. Since broiler morphology pushes their legs further apart [28], with step width increasing markedly in lameness [33], they move their CoM laterally towards the position of each sequential supporting (stance) leg, resulting in the stereotypical side-to-side waddle [7], [9], [14]. Our findings support this since the jungle fowl collectively had the least lateral back displacement and the lame broilers had the most. A reduction in joint mobility, in combination with a morphological requirement for greater lateral body displacement during locomotion, forces broilers to kick out their feet laterally. This was especially noticeable when the non-lame broilers were moving at faster speeds and may explain why they lift their feet so high. It is possible that mechanical restraints resulting from additional body mass and joint inflexibility, or discomfort, may have prevented the lame broilers from lifting their legs to a comparable height, although still higher than the jungle fowl.

Vertical back movement was substantially less than lateral movement for all groups. We hypothesise that the high vertical movement recorded in the broilers was due to a combination of limb stiffness and vaulting of the CoM over the stance limb during support. When viewed from the rear the substantial lateral back displacement arising from the ‘waddle’ causes the central back marker to move in an arc between alternate near-horizontal left and right positions. Vertical excursions are likely to be less in our jungle fowl because sound animals flex their joints at the beginning and end of stance to smooth the transition between stance and swing (reducing energy expenditure and increasing efficiency).

### Asymmetry

As part of this study (unreported here) differences between paired strides (one right and one left) were calculated without valence to assess stride asymmetry. Broilers demonstrated greater paired-stride differences than jungle fowl, indicating a less energy-efficient gait. Non-lame broiler gait also appeared to be more asymmetrical than lame broiler gait, which may suggest limited mechanical gait variability in the latter, although this requires further investigation. Since no record was made regarding the valence of the paired-stride differences we were unable to determine whether the broilers were more dominant on one leg than another. This is a possibility since chicken brains are highly lateralised (e.g. [36]) and left- and right-handed dogs have been identified [37]. Unilateral lameness (although less common than bilateral lameness) is also found within intensive broiler flocks and, although not specifically identified via the current gait scoring system, the extent of unilateral lameness could be readily quantified using this methodology.

### Conclusion

This study has shown that the gait patterns of jungle fowl and broilers can be readily recorded and quantified using a modern motion capturing system following temporary (2 h) feed withdrawal. For the first time, key differences in gait were demonstrated between the ancestral and modern line (short stance phase, low double-leg support, low leg lift, and low back displacement in adult jungle fowl; high double-leg support, high leg lift, and high vertical back movement in non-lame broilers), as well as some similarities (stride length and duration). Further modifications to the ‘normal’ non-lame broiler gait pattern were also identified in lame broilers (short stride length and duration, high lateral back movement), although some changes were not always in the direction anticipated. Some of the gait characteristics demonstrated by the non-lame broilers are likely to be of morphological consequence, due to the birds having adapted to a shift in their CoM. The subsequent modifications observed within the lame birds may be due to additional morphological changes within these birds (lamer birds often tend to be heavier) and/or other factors such as discomfort.

Lameness remains a key welfare issue in commercial broiler flocks. Abnormalities in gait can result from a combination of the pathology and its underlying cause, plus modifications made by the individual to regain mobility. Any attempt to accurately assess whether a gait pattern is due to pain or biomechanical factors is therefore extremely difficult. We envisage a vital future role for this methodology in addressing this question via the investigation of therapeutic agent efficacy in treating broiler lameness (for research purposes). There may also be potential for use in genetic selection programmes for gait improvement since kinematic analysis can detect subtle changes in gait pattern that cannot be easily quantified by visual observation.
